# Apparent diffusion coefficient estimates based on 24 hours tracer movement support glymphatic transport in human cerebral cortex

**DOI:** 10.1038/s41598-020-66042-5

**Published:** 2020-06-08

**Authors:** Lars Magnus Valnes, Sebastian K. Mitusch, Geir Ringstad, Per Kristian Eide, Simon W. Funke, Kent-Andre Mardal

**Affiliations:** 1 Department of Mathematics, University of Oslo, Oslo, Norway; 2 Center for Biomedical Computing, Simula Research Laboratory, Lysaker, Norway; 3 Department of Radiology, Oslo University Hospital - Rikshospitalet, Oslo, Norway; 4 Institute of Clinical Medicine, Faculty of Medicine, University of Oslo, Oslo, Norway; 5 Department of Neurosurgery, Oslo University Hospital – Rikshospitalet, Oslo, Norway

**Keywords:** Neurophysiology, Applied mathematics

## Abstract

The recently proposed glymphatic system suggests that bulk flow is important for clearing waste from the brain, and as such may underlie the development of e.g. Alzheimer’s disease. The glymphatic hypothesis is still controversial and several biomechanical modeling studies at the micro-level have questioned the system and its assumptions. In contrast, at the macro-level, there are many experimental findings in support of bulk flow. Here, we will investigate to what extent the CSF tracer distributions seen in novel magnetic resonance imaging (MRI) investigations over hours and days are suggestive of bulk flow as an additional component to diffusion. In order to include the complex geometry of the brain, the heterogeneous CSF flow around the brain, and the transport over the time-scale of days, we employed the methods of partial differential constrained optimization to identify the apparent diffusion coefficient (ADC) that would correspond best to the MRI findings. We found that the computed ADC in the cortical grey matter was 5–26% larger than the ADC estimated with DTI, which suggests that diffusion may not be the only mechanism governing transport.

## Introduction

Most types of dementia are associated with accumulation of metabolic by-products within the brain. In contrast to the rest of the body, the brain lacks a lymphatic system to clear these by-products. In 2012, a new pathway, called the paravascular pathway, was proposed^1^, which enables efficient brain-wide circulation and clearance. The network of paravascular pathways in the brain was named the glymphatic system as it resembles the lymphatic system in the rest of the body, while the ‘g’ in glymphatic highlights the importance of the supportive glia cells. The paravascular pathways consists of cerebrospinal fluid flowing in parallel with the vasculature in paravascular spaces. These pathways have the potential to facilitate exchange between the cerebrospinal fluid (CSF) and the extracellular fluid deep within the brain.

To what extent and at what scale the glymphatic system accelerates transport compared to extracellular diffusion is still controversial, and several computational modeling studies have dismissed parts of the system at micro-scale. For example, the previous studies^2,3^ suggest that diffusion dominates in the interstitium. Furthermore^4–6^, have found that dispersion in the paravascular spaces adds less than a factor two to diffusion for solute transportation. However, multiple experimental and imaging findings at the micro-level point towards transport being different and faster than diffusion^1,7,8^.

Investigation of the paravascular transport at macro-scale was proposed and tested in a rat’s brain^9^. The procedure involved injecting MRI contrast agent into the CSF and subsequently imaging the transportation of the MRI contrast agent at multiple time-points during a few hours after the injection. The MRI contrast agent worked as a CSF tracer, and was brain-wide in the rat after a few hours. The procedure was tested in humans for the first time in 2017^10^ with acquisition of MRI images repeatedly during 48 hours after the injection and later quantified in a region-specific manner in 2018^11^ in individuals with dementia and controls. Overall, the MRI contrast agent transportation showed a centripetal pattern in all participants, but the MRI contrast agent was more protracted in individuals with dementia compared with controls^11^. It was also noted that the CSF-tracer transport appeared significantly faster than what can be expected from diffusion in simplified planar geometries.

On this background, our purpose in this paper is to explore whether the CSF tracer distribution seen in^11^ can be explained by diffusion alone, as predicted by the seminal work of Syková and Nicholson^12^. We will investigate this hypothesis with finite element simulations of the diffusion process combined with a parameter identification procedure for the apparent diffusion coefficients (ADCs). Thus, we aim to investigate whether we can assess ADC on long time-scales (hours or days), by fitting a diffusion model to the MRI data obtained at multiple time-points when the CSF tracer is propagating through the brain. This includes taking into account the complexity of the folding brain surface by constructing patient-specific geometries. Our approach for the parameter identification is to solve an optimization problem constrained by a diffusion equation with unknown coefficients, where the optimization targets the observed CSF tracer concentrations at 5–6 available acquisitions during 24 hours after CSF tracer injection. We remark that paravascular flow is a micro-scale phenomena with velocities at around 20 *μm*/*s* in paravascular spaces with width around 20 *μm*^7^. Hence, any paravascular bulk flow will not be directly visible with current MRI techniques, but may be indirectly measurable through the estimation of the apparent diffusion coefficient at long time-scales.

An outline of the paper is as follows: In Section 1, we present the methodology of the paper. We start in Section 1.3 with a detailed description of the medical imaging methods relevant for this study. Section 1.4 describes the mathematical models, and the computational methodology for this paper. In Section 2, we will present the results of the study, starting with the MRI analysis in Section 2.1. We continue in Section 2.2 with a synthetic test case, which involves finding robust regularization parameters with a uniform distributed noise added to the images. The construction of the synthetic test case and the concentration estimation can be found in the Supplementary. While in Section 2.3, we present the computed ADC using the MRI images, and compare the values with ADC estimated with DTI. In Section 2.3.1, we present the results of different method to decrease the boundary noise. The results will facilitate the general discussion in Section 3.

## Methods

### Simulation workflow

**Figure 1:**
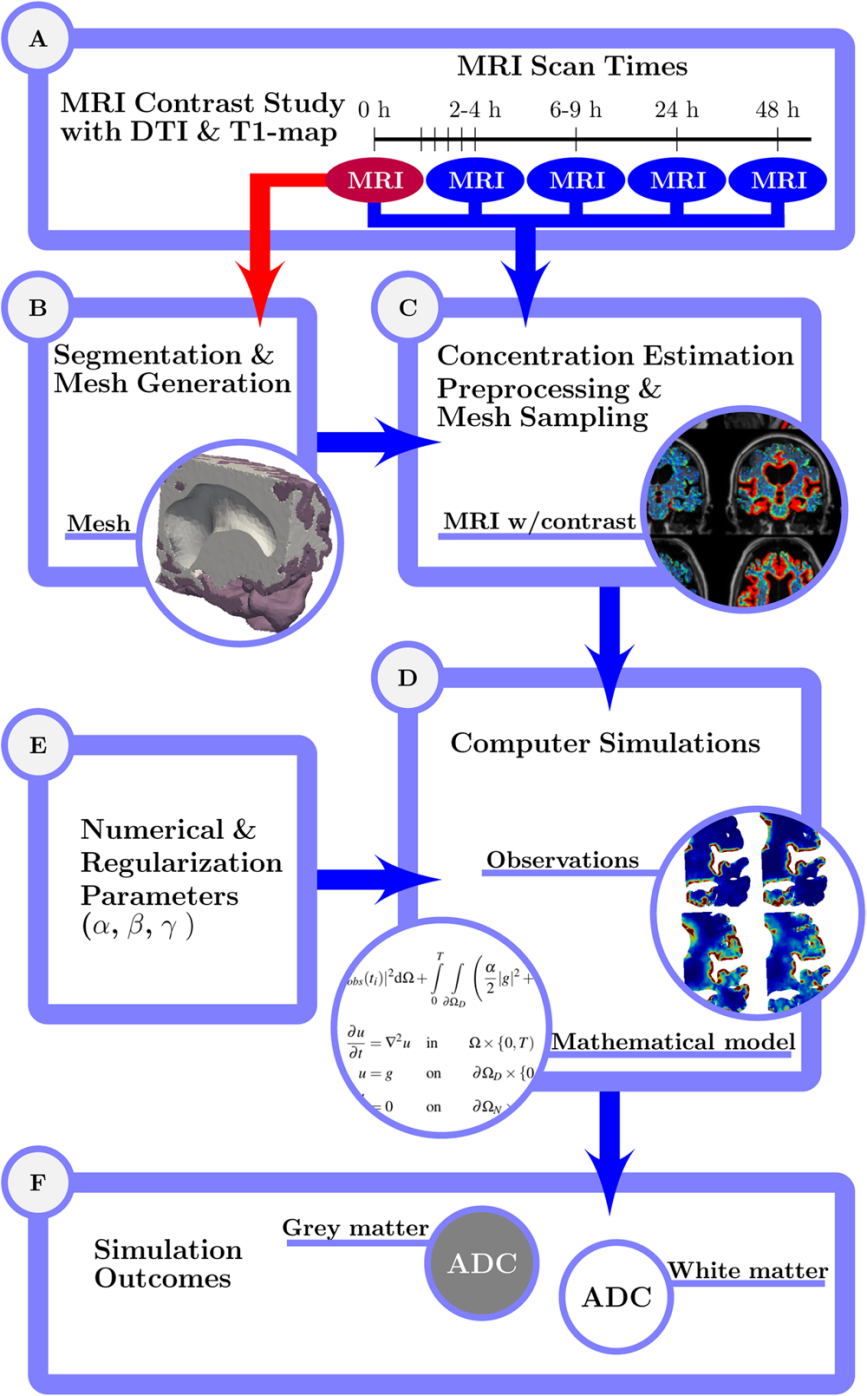
Schematic description of the simulation workflow, outlined through panels A to F.

An overview of the simulation workflow for this paper is outlined in Fig. 1. We obtained MRI data for three subjects, which included MRI images with MRI contrast agent at different times (Box A in Fig. 1). The first MRI image for each subject was segmented and used to construct a subject specific mesh (Box B in Fig. 1). MRI images were used for estimating the CSF tracer concentration for the different times, and were subsequently sampled onto the subject specific meshes (Box C in Fig. 1). The sampled concentrations at the different times were then used with the mathematical model (Box D in Fig. 1). Values for numerical and regularization parameters were inputs for the computation (Box E in Fig. 1). The simulations produced the optimal ADC for grey and white matter to explain the observations for different input parameters(Box F in Fig. 1).

### Approvals and MRI acquisition

The approval for MRI observations was retrieved by the Regional Committee for Medical and Health Research Ethics (REK) of Health Region South-East, Norway (2015/96) and the Institutional Review Board of Oslo University Hospital (2015/1868) and the National Medicines Agency (15/04932-7). The study participants were included after written and oral informed consent. Two of the participants were diagnosed with normal pressure hydrocephalus (NPH1 and NPH2), while one was a reference (REF). The MRI images included 3D T1-weighted volume, sagittal 3D FLAIR, DTI and T1 map for the same patients. All methods were performed in accordance with the relevant guidelines and regulations.

The contrast observations were obtained using a 3 Tesla Philips Ingenia MRI scanner (Philips Medical Systems) with the same imaging protocol settings at all time points to acquire sagittal 3D T1-weighted volume scans. The imaging parameters were as follows: repetition time, “shortest” (typically 5.1 ms); echo time, “shortest” (typically 2.3 ms); flip angle, 8 degrees; field of view, 256 × 256 cm; and matrix, 256 × 256 pixels (reconstructed 512 × 512). We also obtained observation a sagittal 3D FLAIR volume sequence of the same patient, that was taken before the injection of contrast. The main imaging parameters were; repetition time = 4,800 ms; echo time 318 ms; inversion recovery time, 1,650 ms; field of view, 250 × 250 mm; and matrix, 250 × 250 pixels (reconstructed 512 × 512). The T1 map was obtained with a MOLLI5(3)3^13^ sequence with the following imaging parameters; repetition time 2.3 ms; echo time 1.0 ms; flip angle 20; field of view 257 × 257; and matrix 240 × 240 pixels. The DTI acquisition was done with the parameters; repetition time 12171 ms; echo time 60.0 ms; flip angle 90; field of view 240 × 240; and matrix 96 × 96 pixels; b-values 0 and 1000, 16 b-vectors, and slice thickness 2.5 mm.

### MRI analysis

**Figure 2:**
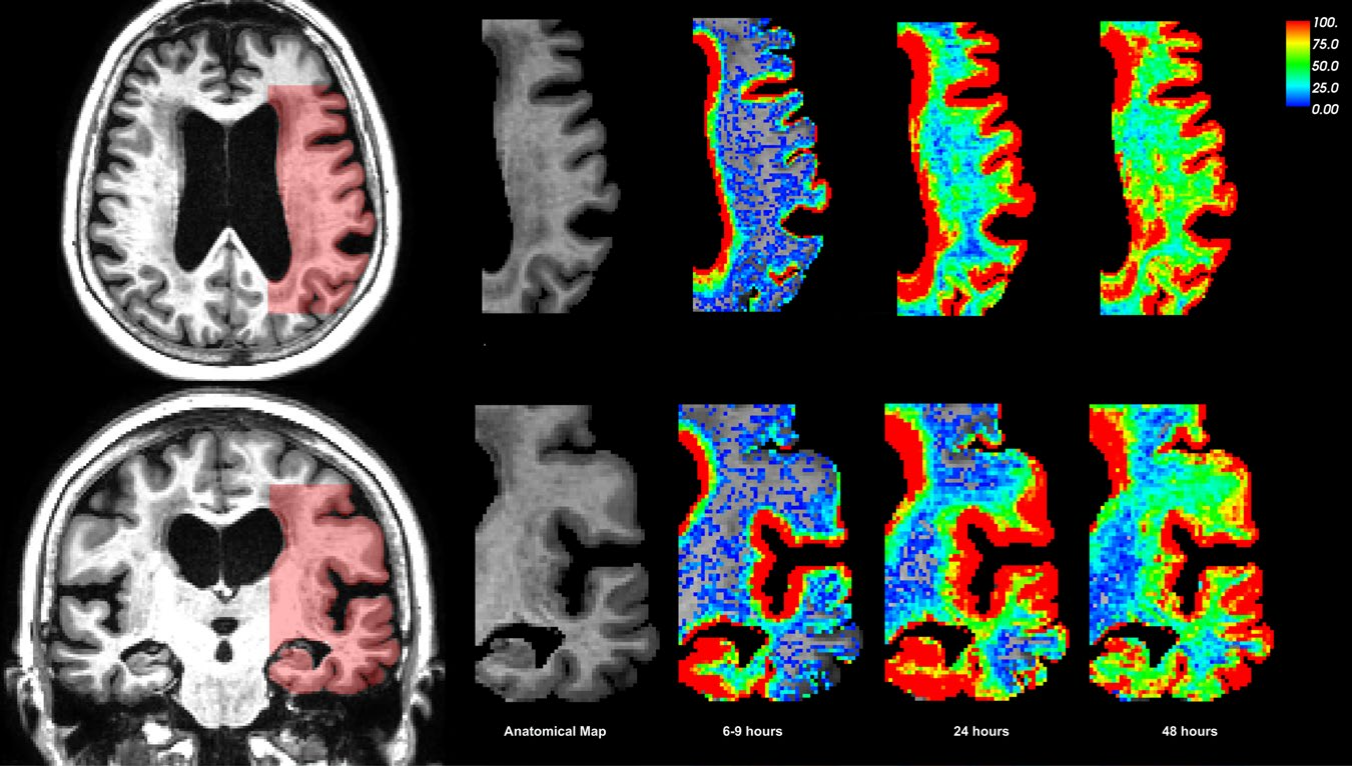
The image shows the percentage change in T1 signal unit ratios from baseline at different observation times in the slice (marked red in the left panel) used in the subsequent analysis in NPH1. The color-bar was restricted to the range (0, 100). The upper row shows the axial slice, and the bottom row shows the coronal slice.

Time sequences of T1-weighted MRI images showing the CSF- and brain enhancement in the subjects during 48 hours after intrathecal administration of 0,5 ml 1 mmol/ml of gadobutrol (Gadovist, Bayer AB, Sweden), was obtained from a previous study^11^. The software FreeSurfer^14–17^ was used to segment and align each of the MRI images, which made it possible to estimate voxelwise signal increase. Figure 2 shows the distribution of MRI contrast agent in a selected region, as a percentage change in MRI signal unit ratios in NPH1. The full data set used in this study (not all shown) consists of 9–10 MRI images, including a baseline MRI image taken before the contrast agent was injected. The MRI scans were obtained at different times distributed over 5 scans within 1–2 and then 4–5 scans over the next 48 hours, see Table 1. We segmented the baseline image with FreeSurfer and aligned the other images to the baseline. The exponential relation between the MRI signal values and the CSF tracer concentration, and the estimation of the concentration for each voxel is documented in the Supplementary Section 1.3. The estimation of the concentration produced images similar to the MRI images, but the values have the unit millimolar (mM). Therefore, we will denote the concentration images as observations to distinguish the concentrations from the MRI image intensities.

**Table 1:** The table shows the number of vertices for each grid, the time step value (Δ*t*), the observation times T_*obs*_ (hours after first MRI with intrathecal administration) of the different subjects and the regularization parameters used.

ID	Mesh cells	Mesh vertices	Δ*t*	T_*obs*_	*α*	*β*	*γ*
(NPH1)	183138	42514 vertices	1 h,2 h	[0.0, 2.1, 61, 24.0, 478]	10^−6^, 10^−4^	1.0, 10.0	0.0, 0.01, 1.00
(REF)	240360	57200 vertices	1 h,2 h	[0.0, 2.1, 41, 5.7, 505]	10^−6^, 10^−4^	1.0, 10.0	0.0, 0.01, 1.00
(NPH2)	260697	61924 vertices	1 h,2 h	[0.0, 2.0, 40, 6.0, 240, 47.5]	10^−6^, 10^−4^	1.0, 10.0	0.0, 0.01, 1.00

**Figure 3:**
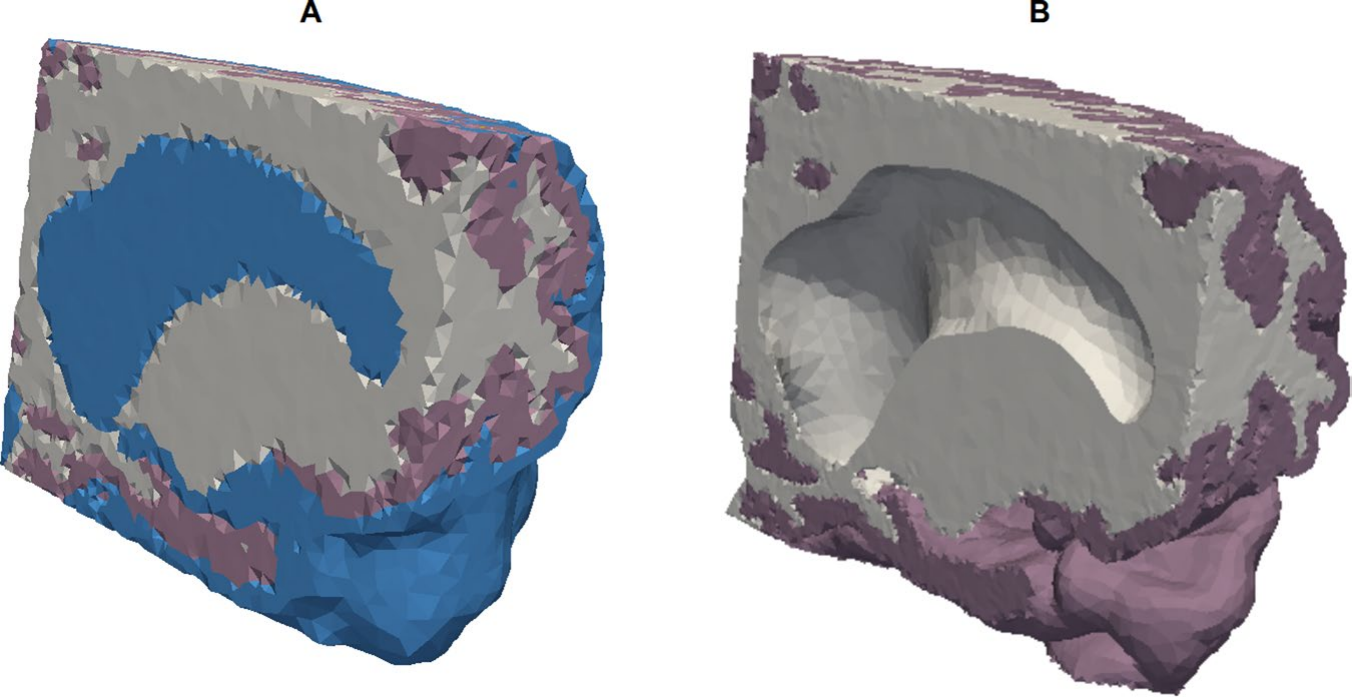
The leftmost image (**A**) shows the mesh created from the baseline MRI image with three domains, while the rightmost image (**B**) shows the mesh created from the baseline MRI image with two domains. The blue domain corresponds to CSF domain, Ω_*CSF*_, the purple domain corresponds to grey matter, Ω_*GM*_, and the white domain corresponds to white matter, Ω_*WM*_. Both images concern NPH1.

The segmentation process also produced polyhedral surfaces of the white and cortical grey matter that were used for mesh construction. We used the Computational Geometry Algorithms Library (CGAL)^18^ to combine the surfaces and construct the mesh with different subdomains. The computational requirement for the resulting mesh was significant, therefore two submeshes were also constructed, see Fig. 3. For the first individual we performed a set of extra tests in order to assess the robustness and accuracy of the computational method. Included here was additional tests where we included the CSF domain in the estimation process. In this case we constructed a three domain mesh (white matter, grey matter and CSF compartment), shown in Fig. 3A, consists of 244,318 tetrahedral cells and 22,057 vertices. For all participants we constructed two domain meshes, shown in Fig. 3B, mesh size and cells are reported in Table 1.

### Mathematical model

The macroscopic extracellular diffusion in the brain can be considered a hindered diffusion with an apparent diffusion coefficient (ADC) depending on the structure of the extracellular space^12^. The relation between the apparent and free diffusion coefficients is defined as

1 $$\lambda =\sqrt{D/{D}_{ADC}}$$

with *λ* denoting the tortuosity of the extracellular space. In order to estimate the ADC involved in the contrast transportation, shown in Fig. 2, we assume that the process can be modeled by a diffusion equation. Then, we constructed an optimization problem with the aim of minimize the difference between the observed and the modeled contrast distribution by optimizing the boundary conditions and the apparent diffusion coefficient. Thus enhanced transportation because of effects such as dispersion would result in an ADC larger than what extracellular diffusion can explain alone. The optimization problem was defined as

2 $$\mathop{{\rm{\min }}}\limits_{D,g}\,\mathop{\sum }\limits_{i\mathrm{=1}}^{n}\,{\int }_{\Omega }\,|u({t}_{i})-{u}_{obs}({t}_{i}{)|}^{2}\,{\rm{d}}\Omega +{\int }_{0}^{T}\,{\int }_{\partial {\Omega }_{D}}\,\left(\frac{\alpha }{2}|g{|}^{2}+\frac{\beta }{2}{|\frac{\partial g}{\partial t}|}^{2}+\frac{\gamma }{2}|\nabla g{|}^{2}\right){\rm{d}}\Omega {\rm{d}}t$$

subject to

3 $$\begin{array}{lllll}\frac{\partial u}{\partial t} & = & D{\nabla }^{2}u & {\rm{in}} & \Omega \times \mathrm{\{0,}T)\\ u & = & g & {\rm{on}} & \partial {\Omega }_{D}\times \mathrm{\{0,}T)\\ \frac{\partial u}{\partial n} & = & 0 & {\rm{on}} & \partial {\Omega }_{N}\times \mathrm{\{0,}T)\end{array}$$

Here, *u*[mM] is the simulated, time-varying CSF tracer distribution, *D*[mm^2^/s] is the ADC, *g*[mM] is the boundary condition, Ω is the domain, and *T*[h] is the final simulation time. We assume that the domain Ω consists of three sub domains, each with a different ADC. We denote the CSF (subarachnoid and lateral ventricle) domain as Ω_*CSF*_, the grey matter as Ω_*GM*_ and the white matter as Ω_*WM*_. The ADC was assumed to be constant within the CSF, grey and white matter but each region may have different values. The *α*, *β* and *γ* parameters are non-negative regularization parameters and *u*_*obs*_[mM] are the concentration distribution at time-points *t*_*i*_[h]. Spacial regularization parameter *α* enforces smoothness on the boundary by minimizing the concentration, i.e. high value of *α* will give less concentration in the optimal solution. Temporal regularization parameter *β* enforces smoothness in time on the boundary, i.e. high value of *β* will give a smoother concentration curve in time. Gradient regularization parameter *γ* enforces continuity between adjacent concentrations at the boundary, i.e. high values of *γ* will give smoother concentration values at the boundary.

#### Boundary conditions

For the three domain geometry (with grey and white matter, and CSF compartment), the Dirichlet boundary condition Ω_*D*_ was only applied on the outward facing boundary of the CSF domain, *∂*Ω_1_. Homogeneous Neumann conditions Ω_*N*_ were applied on the remaining boundaries.

**Figure 4:**
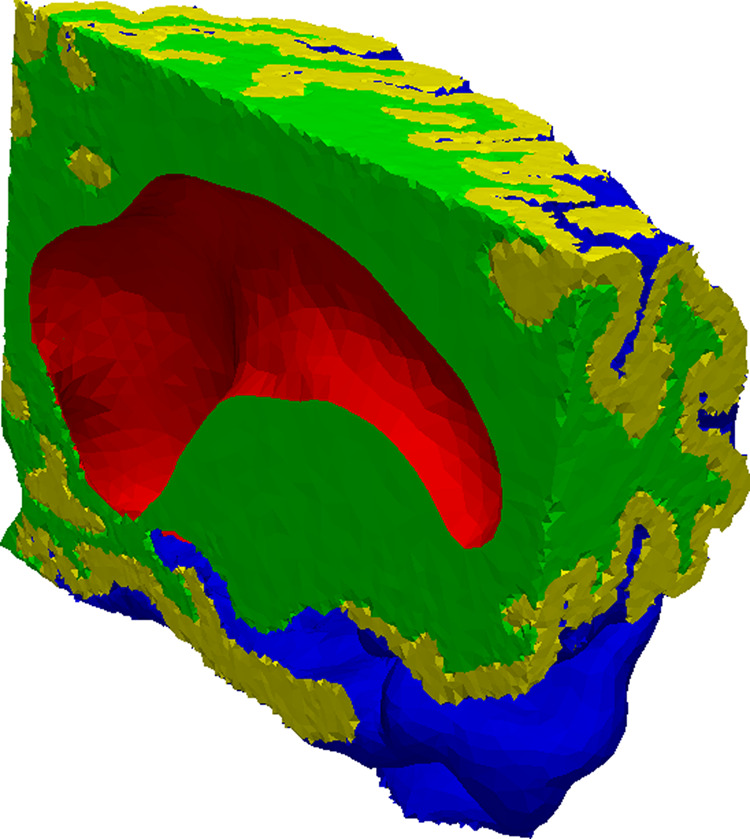
The images shows the different boundary values on the mesh, with each color representing a unique boundary.

The implementation of the gradient regularization *γ* for the case containing only grey and white matter required that the outward facing boundary was decomposed in different regions to avoid the boundary values being continuous at the interface between CSF, grey and white matter. We decomposed the boundary as seen in Fig. 4, with the red and blue boundary adjacent to the CSF. We defined the red boundary *∂*Ω_*r*_ and the blue boundary *∂*Ω_*b*_ as Dirichlet boundaries Ω_*D*_, while the green and yellow were Neumann boundaries Ω_*N*_. The regularization parameter *γ* was subsequently set to be non-zero in (2). We initially tested gradient regularization with the same parameter *γ* on both boundaries, but the different distribution of tracers on the boundaries made it difficult to find an adequate value for *γ*. This may be attributed to the fact that the concentration in the lateral ventricles were more uniform than that in the SAS, so we defined *γ* as

4 $$\gamma =\{\begin{array}{lll}0.01\tilde{\gamma } & \in  & \partial {\Omega }_{b}\\ \tilde{\gamma } & \in  & \partial {\Omega }_{r}\end{array}$$

with $\tilde{\gamma }$ as the referenced, i.e. mentioned in the text, regularization parameter.

#### Synthetic test case with a manufactured solution for NPH1

In order to assess the robustness and accuracy of the methodology of ADC estimation via PDE constrained optimization we constructed a synthetic test case with a known, manufactured solution for the first participant. The setup for the numerical tests can be found in the Supplementary Section 1.1. In the case of three domains we varied *α* ∈ (10^−6^, 10^−2^) and *β* ∈ (10^−6^, 10^−2^). In the case of two domains *α* ∈ (10^−6^, 1), *β* ∈ (10^−4^, 10^2^) and *γ* ∈ (10^−4^, 1.0).

We tested the noise susceptibility with a uniform distributed of noise. This was done by adding noise in the range of (−*n*_*amp*_, *n*_*amp*_) to the observation after loading, i.e. each vertex. We tested the noise with *n*_*amp*_ ∈ (0.03, 0.15, 0.30, 0.475), compared to the maximum values for the manufactured solution being in the range (0.3,1.3) for all times. We used the observation with the added noise to compute and confirm the expected SNR, since there was a finite number of vertices. The variation in the number of observations was tested with 5,10 and 20 evenly spaced observations over the course of 24 hours combined with 10 times steps, 20 time steps and 40 time steps.

### CSF tracer distribution reconstruction from MRI data of NPH1

The MRI data consisted of scans at times *t*_*i*_ that were distributed in the 48 hours time frame, with the first observation with tracer occurring 1–2 hours after the injection. This was followed by 4 observations within the first hour, and we observed no visible change in the tracer distribution for these observations. Therefore, we used observation times listed in Table 1 for the computation of the ADC values.

#### Preprocessing of concentrations of gadobutrol at ventricular and subarachnoid surfaces for NPH1

**Figure 5:**
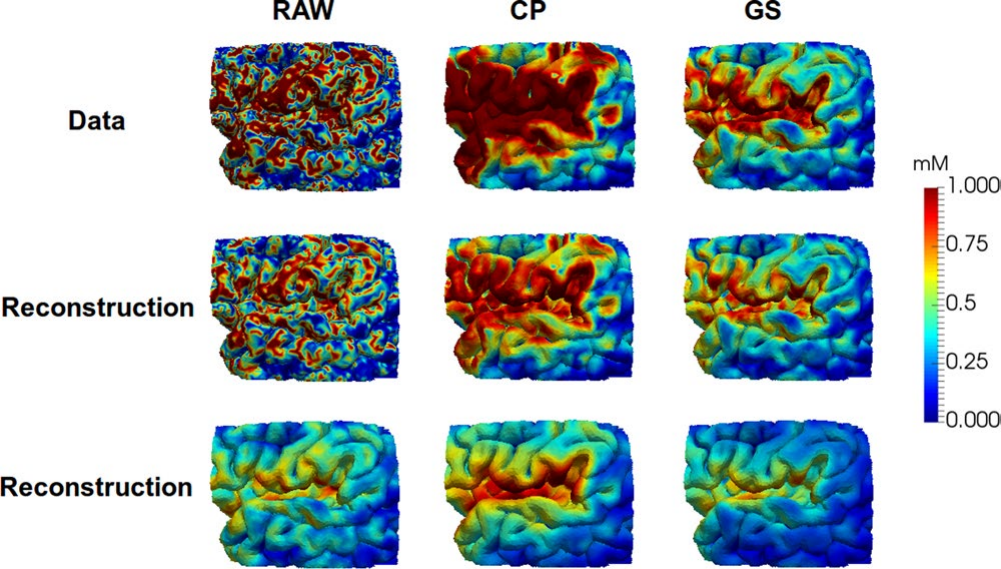
The images shows the SAS boundary after 12 hours. The upper row shows the observations with following preprocessing left to right: Raw observations, projection of CSF value onto the boundary, Gaussian smoothing. The middle row shows the corresponding states with the regularization parameters *α*, *β*, *γ* = (10^−6^, 1.0, 1.0). The bottom row shows the corresponding states with the regularization parameters *α*, *β*, *γ* = (10^−6^, 100.0, 100).

**Figure 6:**
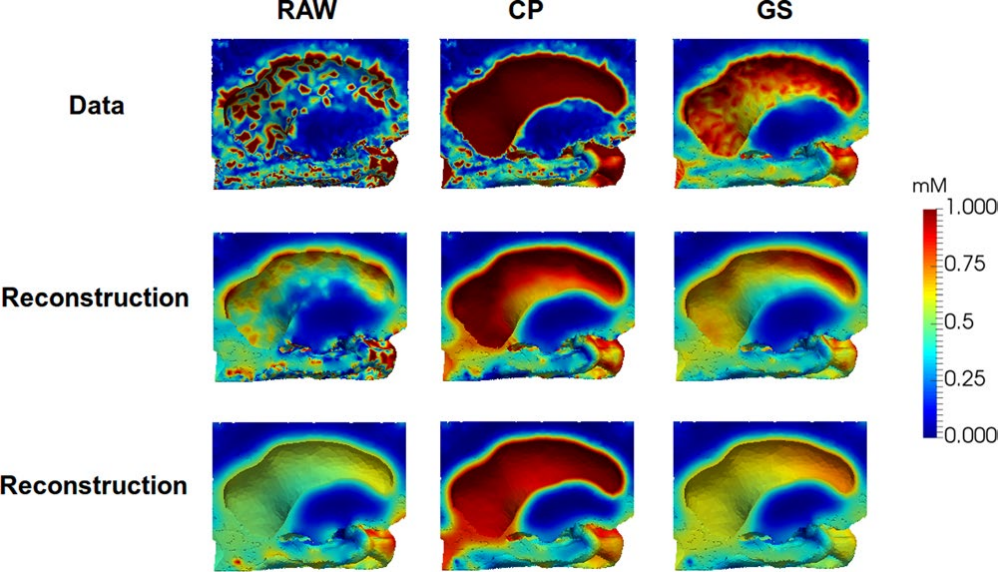
The images shows the ventricular wall after 12 hours in NPH1. The upper row shows the observations with following preprocessing left to right: Raw observations, projection of CSF value onto the boundary, Gaussian smoothing. The middle row shows the corresponding states with the regularization parameters *α*, *β*, *γ* = (10^−6^, 1.0, 1.0). The bottom row shows the corresponding states with the regularization parameters *α*, *β*, *γ* = (10^−6^, 100.0, 100).

High frequency concentration changes was observed at the boundaries of our mesh, see top row of column A in Figs. 5 and 6, which can be interpreted as sampling errors. Such errors may be caused by noise in the MRI data, errors in the segmentation, the segmented polyhedral surfaces which typically cut voxels, miss-alignment between different observations, and by the inaccuracy of sampling discontinuous voxel data. Therefore, we investigated two approaches to reduce high frequency components at mesh boundaries:

- A projection of the segmented CSF gadobutrol concentrations directly at the ventricle and subarachnoid surfaces (CP),
- A Gaussian smoothing (GS) procedure.

In detail, the CP method was implemented by finding the voxel corresponding to each boundary vertex on Ω_*b*_ and Ω_*r*_ using an affine transformation matrix in FreeSurfer. Then, for each voxel, we computed the average of all surrounding voxels with CSF segmentation mark in a 7 × 7 × 7 matrix. The average values were then used at the corresponding boundary vertex in the computations. In order to estimate the CSF concentration, we assumed that the T1 value corresponding to CSF was 3000 ms. Finally, for the GS method we used the Gaussian smoothing function found in the python-module scipy^19^, and applied the smoothing to all voxel in the observations estimated from MRI images. The standard deviation of the Gaussian distribution was set to 1.5 mm^2^ compared to MRI voxel length of 1.0 mm. In addition to the CP and GS methods, we also use the raw data without any preprocessing. This method is referred to as RAW.

We performed the simulations with the regularization parameters *α* ∈ (10^−6^, 10^−4^), *β* ∈ (1.0, 10), *γ* ∈ (0.0, 0.01, 1.0). Additionally, we ran simulation with *β* = 100 and *γ* = 100 to obtain simulations that clearly showed differences in the concentration distributions on the boundary.

#### Comparison with data obtained from DTI analysis for all participants

Based on the investigation of NPH1, the parameters of 1 was used to assess the ADC of REF and NPH2. Furthermore, we compared the ADC computed by solving (2)-(3) with ADC values obtained from the DTI image of the same patients. We remark that a direct comparison with DTI is not possible because DTI measures the ADC of water. Therefore, we used the DTI to estimate the tortuosity in grey and white matter, which together with the free diffusion coefficient of gadobutrol and (1) can be used to approximate ADC for gadobutrol, details and references are found in the Supplementary Section 1.4. The difference between simulations and observations is quantified as the relative *l*_2_ norm of the difference (RL2ND) between the simulated and observed contrast, i.e., ${\sum }_{i}\,||u({t}_{i})-u{({t}_{i})}_{obs}{||}_{{l}^{2}}/||u{({t}_{i})}_{obs}{||}_{{l}^{2}}\mathrm{}.$

## Results

### MRI analysis

Statistical analysis was performed to determine the signal to noise ratio (SNR) for each observation, see Table 2. The noise was estimated by taking the standard deviation over an interval containing the largest peak of the concentration distribution. Then, the SNR was estimated by taking the average over the same interval divided by the noise. In Table 2, we see that the SNR is quite low indicating low average concentration and/or high standard deviation. Note that we expect low concentration in the first 1–2 hours as the CSF tracer has not sufficiently enriched the brain parenchyma yet. This explains the low SNR in the first hours.

**Table 2:** The table shows the SNR range in grey and white matter over two time intervals for each participant.

ID	GM SNR (0–1 h)	WM SNR (0–1 h)	GM SNR (2–48 h)	WM SNR (2–48 h)
(NPH1)	0.3–0.5	0.1–0.7	0.5–1.8	0.6–1.7
(REF)	0.3–0.8	0.3	0.5–1.3	0.4–1.8
(NPH2)	0.3–0.5	0.5–1.0	0.7–1.5	0.8–1.9

### Assessment of accuracy and robustness on a synthetic test case for NPH1

For the first geometry, involving CSF, cortical grey and white matter, we ran a series of 448 tests with different regularization parameters *α* and *β* using the manufactured solutions with observations every 2.4 hours over the course of 24 hours. It was found that for *α* ∈ (10^−6^, 10^−2^) and *β* ∈ (10^−6^, 1.0), the error in the ADC for CSF, grey and white matter was less than 5%. For combinations with *β* = 10^2^, the smallest error was 63.6% in the CSF, 10.8% in grey matter and 3.5% in white matter. While for *α* = 1.0, the smallest error was 160.2% in the CSF, 23.2% in grey matter and 8.9% in white matter. Second, the robustness of the parameter identification process with respect to noise in the data was investigated. The noise values were randomly obtained from a uniform distribution in the range (−0.3,0.3), where 0.3 equaled the maximum initial value of the manufactured solution and was 23% of the manufactured solution at its max. Again, for *α* ∈ (10^−6^, 10^−2^) and *β* ∈ (10^−6^, 1.0) the error in the ADC in CSF was less than 23% and less than 9.7% in grey and white matter.

For the second geometry, including only cortical grey and white matter, we ran a selection of 186 tests to ensure that the results were consistent. The boundary conditions were applied to the boundaries of the SAS and lateral ventricle. In the range *α* ∈ (10^−6^, 10^−2^) and *β* ∈ (10^−4^, 1.0), the error was less than 4.2% for the ADC in both grey and white matter.

The noise susceptibility was tested with the addition of the regularization parameter *γ* ∈ (0.0, 10^−2^, 1.0), which enforces smoothness at the boundary. We computed the error on the SAS boundary and on the lateral ventricle boundary, and it was observed that *γ* did not contribute to a lower error in the ADC, but decreased the boundary error with a few percentage on average. In the range *α* ∈ (10^−6^, 10^−4^) and *β* ∈ (10^−4^, 1.0), we had maximum error of 7.0% in the grey matter and 3.1% in the white matter with noise randomly obtained from the uniform distribution range of (−0.3,0.3). The computed the SNR of 0.74 for first time step, and increased to the maximum of 6.3.

The synthetic test case revealed that the second geometry, involving only the estimation of ADC in grey and white matter was the most accurate method. Hence, in the following, only the second geometry was used.

### CSF tracer distribution reconstruction from MRI data for NPH1

**Figure 7:**
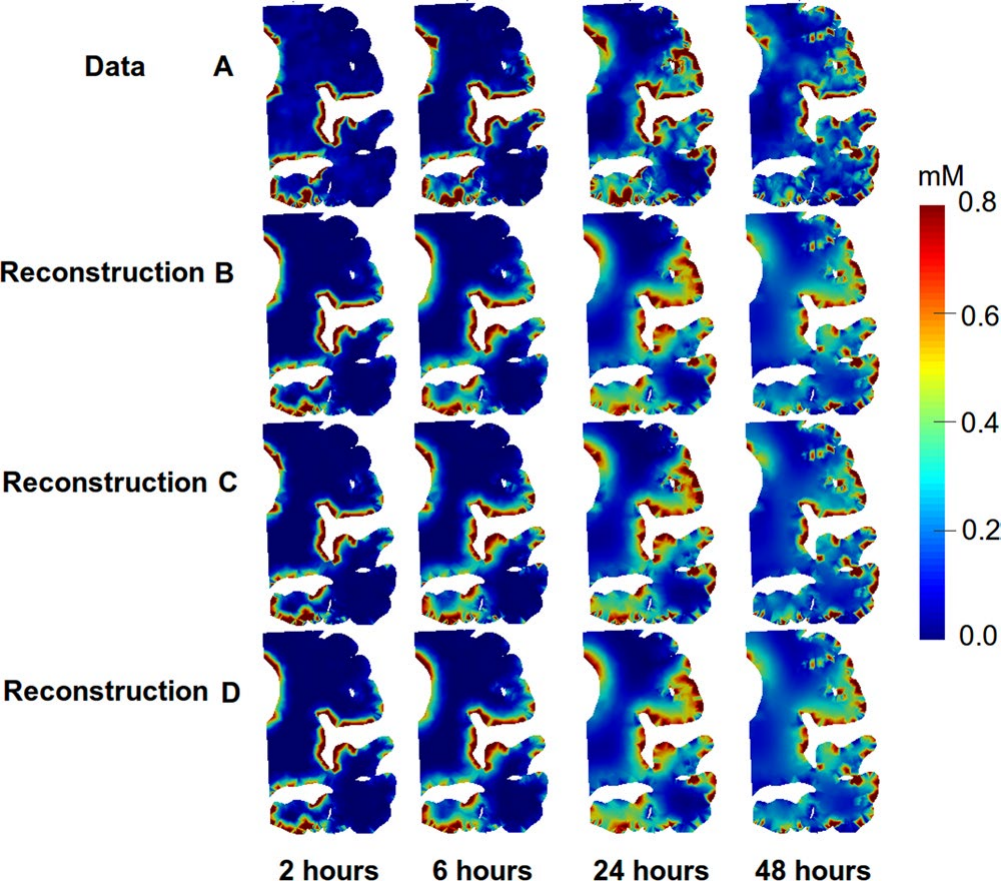
The image displays observations from MRI images and computational reconstruction of the same observations at observational time-points, i.e. 2 hours, 6 hours, 24 hours and 48 hours after the first MRI with administration of CSF tracer in NPH1. Row (**A**) shows the observations estimated from MRI images. Row (**B**) shows the reconstructed observations with *α* = 0.0001, *β* = 10.0, *γ* = 1.0 and 48 time steps. Row (**C**) shows reconstructed observations with *α* = 10^−6^, *β* = 1.0, *γ* = 0.01 and 48 time steps. Row (**D**) shows reconstructed observations with *α* = 10^−6^, *β* = 10.0, *γ* = 1.0 and 48 time steps. The color-bar was restricted to the range (0.0–0.8).

**Figure 8:**
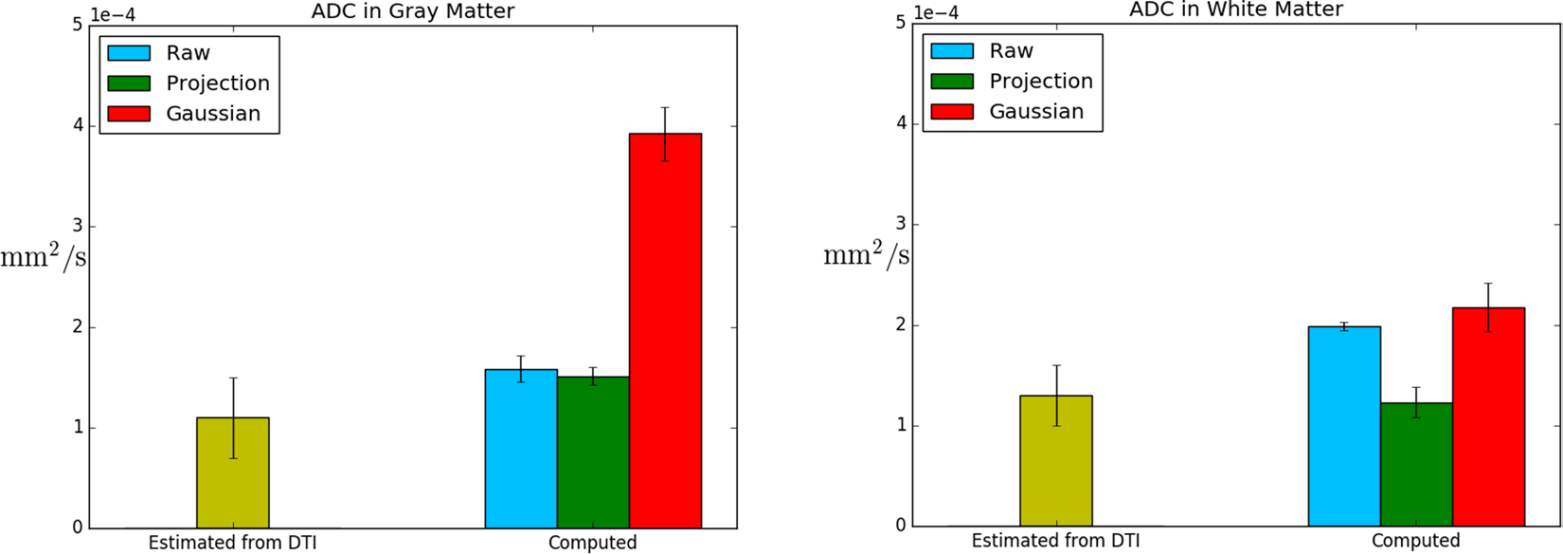
The images shows the average ADC estimated with DTI and the computed ADC in grey and white matter for different sampling methods and regularization parameters *α* ∈ (10^−6^, 10^−4^), *β* ∈ (1.0, 10.0), *γ* ∈ (0.0, 0.01, 1.0) and number of time steps *k* ∈ (24, 48) in NPH1. The error bars show the standard deviation.

In the synthetic case we managed to reproduce the ADC within 7% error for a wide range of parameters, *α* ∈ (10^−6^, 10^−2^), *β* ∈ (10^−4^, 1.0), and *γ* ∈ (0.0, 10^−2^, 1.0). The same parameters were then used to compute the optimal ADC for the CSF tracer estimated from MRI images. A few of the reconstructions are shown in Fig. 7. It is clear that our model assumption of an underlying diffusion equation is adequate, that is; from a visual point of view the observed data in Fig. 7, row A, is reconstructed accurately for the various different regularization parameters Fig. 7, row B-D. We computed the average ADC over the range of regularization parameters to be 0.57 ± 0.05 mm^2^/h in grey matter and 0.72 ± 0.02 mm^2^/h in the white matter, which respectively corresponds to 1.6 ± 0.1 × 10^−4^ mm^2^/s and 2.0 ± 0.1 × 10^−4^ mm^2^/s, shown in Fig. 8.

#### Preprocessing of concentrations of gadobutrol at ventricular and subarachnoid surfaces for NPH1

The three different reconstructions with RAW, CP and GS methods are illustrated in Figs. 5 and 6 for some different regularization parameters. As can be seen in RAW column in Figs. 5 and 6 the boundary gradient regularization *γ* caused the concentration to be more uniform on the boundary and in particular for the high value *γ* = 100. The CP method results in similar values for most of the ventricular boundary. The resulting ADC gadobutrol values for RAW, CP and GS are shown in Fig. 8 together with the gadobutrol ADC value estimated from DTI. We computed the percentage difference for each computed ADC and compared with ADC estimated from DTI. For the CP method, the average difference was 37 ± 8% in grey matter, −5 ± 11% in white matter and for the GS method, the average difference was 250 ± 22% in grey matter, 68 ± 18% in white matter.

#### Comparison with data obtained from DTI analysis for all participants

The median ADC for water in the DTI was estimated for each participant, and shown in Table 3. The free diffusion coefficient for gadobutrol was approximated to be 3.8 × 10^−4^ mm^2^/s in the Supplementary Section 1.4. This gives an estimate median gadobutrol ADC in grey and white matter as listed in Table 4. This estimation assumed that the tortuosity was independent for molecules with mass lower than 1 kDa. In all subjects the estimated ADC was higher than the corresponding ADC values in DTI, i.e. 5–26% in grey matter and 29–82% in white matter.

**Table 3:** The table shows the median DTI values for water ADC in grey and white matter for each participant, and the corresponding tortuosity based on (1).

ID	ADC GM	ADC WM	*λ* GM	*λ* WM
(NPH1)	1.0 ± 0.3	0.9 ± 0.3	1.72	1.86
(REF)	1.1 ± 0.3	0.8 ± 0.2	1.66	1.91
(NPH2)	1.1 ± 0.3	0.8 ± 0.3	1.62	1.89

The ADC unit is 10^−3^ mm^2^/s.

**Table 4:** The table shows the ADC of gadobutrol, derived from DTI and simulation (SIM), for the CSF tracer and three different data sets.

ID	DTI GM	DTI WM	SIM GM	SIM WM	DIFF. GM	DIFF. WM	*RL*2*ND*
(NPH)	1.3 ± 0.4	1.1 ± 0.3	1.6 ± 0.1	2.0 ± 0.1	23 ± 10%	82 ± 4%	0.28
(REF)	1.4 ± 0.4	1.1 ± 0.3	1.7 ± 0.1	1.4 ± 0.1	26 ± 7%	30 ± 2%	0.27
(NPH)	1.4 ± 0.4	1.1 ± 0.3	1.5 ± 0.1	1.4 ± 0.1	5 ± 5%	29 ± 12%	0.32

The ADC unit is 10^−4^ mm^2^/s, and the standard deviations for the simulations were estimated from the ensemble of computed values. The right most column reports the relative l2 norm of the differences between simulations and observations (RL2ND).

## Discussion

The glymphatic system proposes that the paravascular network facilitates brain-wide transportation by micro-scale bulk flow. Several mathematical modeling studies have been performed at the micro-level, but to our knowledge, our current study is the first study to investigate this process based on human imaging data at the macro-level. Our investigations are based on the distribution of gadobutrol in CSF and brain tissue up to 48 hours after intrathecal injection. Our results here are supportive of the glymphatic system in the sense that the ADC estimated from the gadobutrol distribution is higher than corresponding numbers obtained from DTI. Hence, our study suggests that at longer time scales, the slow glymphatic system has an impact. The estimated ADC is however, much closer to corresponding DTI values than our previous estimates with diffusion in planar geometries^11^.

**Figure 9:**
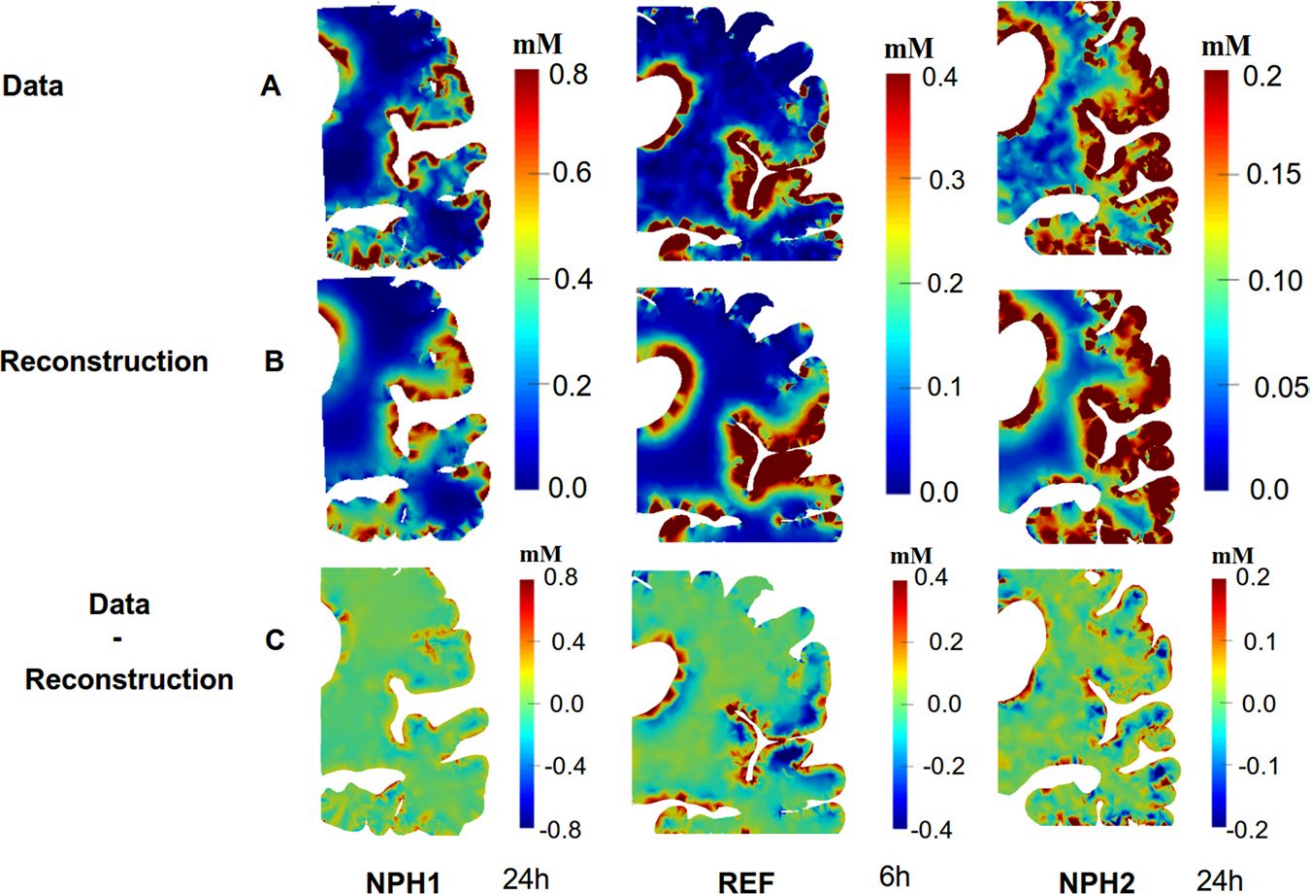
The images shows the both the data and reconstructions after 24 h, 6 h and 24 h for different data sets (NPH1, REF, NPH2).

To the authors’ knowledge this is the first effort in which the long-term transportation of CSF-tracers is assessed by PDE constrained optimization employing CSF-tracer concentrations at multiple time-points during 48 hours. The estimation process is challenging and there are several limitations that should be investigated further. We assumed that the white matter was isotropic, however, it is well-known that the white matter is anisotropic. The main reason for our choice was that introducing anisotropy would result in a model free variables in every point, representing local direction and anisotropy. The ill-posed structure of the problem would require additional regularization, requiring substantial parameter tuning, and consequently result in less predictability. Furthermore, it can be seen in Figs. 7 and 9 that the reconstruction is quite good and the difference between the simulations and observations is around 0.3 on average (i.e. in the *l*_2_-norm.), dominated by high frequency components. Some of the high frequency differences should or could be attributed to noise as there is SNR relatively small. We remark that assuming that CSF was governed by a diffusion process yielded a much larger error in the synthetic test case and this assumption should probably be avoided.

In our approach we have employed the FreeSurfer toolkit to segment and register grey and white matter surfaces. FreeSurfer provides segmentations with sub-voxel accuracy, which means that at the boundaries towards the ventricles and subarachnoid space, the surface boundary is usually in the interior of a voxel rather than at the voxel boundaries. The consequence is that the raw data appear to have noisy image intensities at the boundaries which cut voxels. For this reason, we investigated two different approaches to interpret the data at the boundaries, in addition to using the raw data itself in detail for NPH1. We observed in Fig. 8 that the GS method increased the computed ADC with approximate 250 ± 22% in grey matter, while the CP method corresponded best with −5 ± 11% difference to the ADC estimated with DTI in white matter. However, it should be noted that the CP method imposes concentration values in the CSF onto the tissue boundaries, not accounting for the transverse propagation of MRI contrast agent through a membrane, like the pia mater or the endothelial layer of the ventricles. Hence, potentially, an unnaturally large concentration gradient at the boundary that is caused by a partial barrier rather than the white matter itself may have resulted in the low ADC seen when using this method. Which of the methods that best depicted the actual boundary concentration is unknown and would need to be determined by phantom studies. Another limitation is that we have not yet been able to assess the diffusivity of gadobutrol and have have relied on literature values for the free diffusion coefficient of a similar mass molecule as a substitute for gadobutrol. However, it should be noted that even though the concentration values appear noisy at the boundaries, Fig. 5, the interior reconstructions appear accurate, Fig. 7.

Concerning the imaging, a current limitation is that standard T1 weighted MRI images have higher resolution and SNR than corresponding T1-maps. In detail, the intrathecal contrast enhanced T1 weighted volume scans had 1.0 mm slice thickness, while the T1-map slice thickness was 4.0 mm and DTI slice thickness was 2.5 mm. This means that the calculation of the concentration at boundaries can suffer from mismatch of tissue and CSF. Furthermore, the T1-map sequence is designed to estimate the T1 times in tissue, and therefore does not give accurate values for the CSF. The average T1 relaxation time for the CSF in left lateral ventricle was 181 ± 349 ms, compared to the value 1000–5500 ms that can be found in the literature^20^ and we therefore used literature values to compute the concentration in the CSF. The reason for the underestimation is that the duration between pulses in the T1-map sequence is to short for the CSF relaxation^21^ and that a different protocol should be used for the CSF. We also estimated the T1 relaxation time for grey and white matter to be 1200 ± 271 and 819 ± 180 ms, which compares better with the literature values ranging 1470–1800 ms in grey and 1084–1110 ms in white matter^22^.

In this study, involving three participants, the simulation based ADC for gadobutrol on the time-scale of 24–48 hours was found to be 1.5 − 1.7 × 10^−4^ mm^2^/s in cortical grey matter and 1.4 − 2.0 × 10^−4^ mm^2^/s in white matter. The corresponding ADC of water in grey and white matter ranged 1.2 − 1.3 × 10^−3^ mm^2^/s and 1.1 − 1.6 × 10^−3^ mm^2^/s, and the tortousity is 1.5–1.6 in grey matter and 1.4 − 1.7 in white matter. A tortousity of around 1.6, but with some variation has been reported normal in brain tissue in several different species and with several different techniques^12^. For instance, using radiotracer in caudate nucleus the tortousity of the extra-cellular matrix varied from 1.50 in a cat to 1.64 in a monkey, using sucrose and Ca-EDTA, respectively. Corresponding ADC of water for the cat and monkey would be 1.3 and 1.1, respectively. Using DTI, the ADC in young and healthy subjects is around 0.78 − 1.09 × 10^−3^ in the cortical grey matter and 0.7 − 0.9 × 10^−3^ mm^2^/s in white matter^23^. Corresponding ADC values for water, based on DTI, in our study was in the range 0.8–1.1 × 10^−3^ mm^2^/s. Although our values match with the DTI values of this study, the grey and white matter seem to be in the upper range. This can be related to the fact that subjects with dementia typically have higher ADC values, less anisotropy and greater variation in the white matter^24^. Furthermore, tissue damage/change can cause the tortuosity to be higher, as reported for Alzheimer’s disease^25^ and ischemia^26^. The participants in this study have neurological diseases, which can have an effect on the extracellular space. Corresponding ADC values for gadobutrol, c.f. Table 4, show that the ADC based on the simulations are 5–26% higher than corresponding values predicted by DTI for gadobutrol (by calculating the tortousity based on the ADC of water). A methodological limitation is the fact that we were not able to include the anisotropy of the white matter in the optimization process. Furthermore, the DTI based ADC of water involves both hindered and restricted diffusion representing both intra-cellular and extra-cellular processes^27^ and we used a standard DTI protocol rather than one that targets extra-cellular diffusion. Alternative imaging, like restriction spectrum imaging (RSI)^28^ or intravoxel incoherent motion imaging (IVIM)^29^ may enable a better separation of the different processes.

Previous mathematical modeling studies at the micro-level^2,3^ suggest that diffusion dominates in the interstitium. However, diffusion depends on molecular size as described by the Stokes-Einstein equations and large molecules are transported slower than small molecules^30^. Convective flow of solutes, on the other hand, is independent of the molecular size. Furthermore, transport has been reported to be independent of molecular sizes^31^, a fact that suggests convective transport. In fact, convective velocities of 0.8–4 × 10^−3^ mm/s has been demonstrated or estimated^8,32–34^, indicating that the solute transport would be dominated by convection for large molecules, whereas similar to diffusion for smaller molecules, such as water. Gadobutrol is in this context a molecule of moderate size, i.e., 604 Da, and hence not ideally suited for the study of bulk flow for larger molecules such as A*β* (4.5 kDa) or CSF-*τ* (45 kDa). In fact, gadobutrol is predicted to have a Pechlet number less than one^2,34^ which from these micro-level studies would imply that the distribution of gadobutrol is governed mainly by diffusion.

Studies^4–6^ have found that dispersion in the paravascular spaces adds less than a factor two to diffusion for solute transportation. However, all these studies were done with modeling that was on the micro-scale over shorter time periods. To the authors’ knowledge, the only other study^35^ that has considered macroscopic modeling on the time-scales of hours and days, where uncertainties representing both variations in ADC and paravascular velocities where modelled with extensive testing using Monte Carlo methods. They found that, in particular, the CSF tracer distribution within the deep white matter found in^11^ could not be explained by diffusion alone. Recent high-resolution MRI imaging of paravascular spaces in a rat’s brain points towards significant contributions from white matter paravascular spaces connected to the ventricles^36^. It should also be mentioned that the permeability of the white matter is several orders of magnitude higher than the grey matter^37,38^ and may as such be more susceptible to bulk flow than grey matter.

In conclusion, we computed that the ADC in grey matter and found that the ADC was somewhat larger than estimates based on DTI alone. Thus, indicating that there is potential for enhanced solute transportation in the brain over a longer time period. There are, however, a number of uncertainties that needs to be taken into account, for instance the resolution of DTI and T1 mapping.

## Supplementary information

Supplementary Information.

## Data Availability

The datasets analyzed in the current study are available from the corresponding author upon request.
