# Supplementary material for: Apparent diffusion coefficient estimates based on 24 hours tracer movement support glymphatic transport in human cerebral cortex
Source: Sci Rep. 2020 Jun 8;10:9176. doi: 10.1038/s41598-020-66042-5 (PMC7280526; doi:10.1038/s41598-020-66042-5)
Supplement: Supplementary file 1 — Supplementary Information. [file 41598_2020_66042_MOESM1_ESM.pdf]

Supplementary Information for "Apparent diffusion coefficient estimates based on 24 hours tracer movement support glymphatic transport in human cerebral cortex"

Lars Magnus Valnes[1], Sebastian K. Mitusch[2], Geir Ringstad[3], Per Kristian Eide[4,5], Simon W. Funke[2], and Kent-Andre Mardal[1,2,*]

[1]Department of Mathematics, University of Oslo, Norway
[2]Center for Biomedical Computing, Simula Research Laboratory, Lysaker, Norway
[3]Division of Radiology and Nuclear Medicine, Department of Radiology - Rikshospitalet, Oslo, Norway
[4]Institute of Clinical Medicine, Faculty of Medicine, University of Oslo, Oslo, Norway
[5]Department of Neurosurgery, Oslo University Hospital – Rikshospitalet, Oslo, Norway
[*]kent-and@math.uio.no

1 Supplementary

1.1 Constructing the synthetic solutions to assess accuracy and robustness.

Below we will discuss the parameter identification and its sensitivity with respect to the regularization parameters, noise, number of observations and time-resolution of the forward model. The manufactured observations used in (2) were obtained by forward computation of (3) with the Dirichlet boundary condition defined as

$$g(t) = 0.3 + 0.167t - 0.007t^2 \qquad \text{for } 0 \leq t \leq 24. \tag{S1}$$

The initial condition was set to 0 everywhere, the time step was $dt = 0.24$, and the diffusion coefficients were selected to be

$$D_{\Omega_1} = 1000.0, \quad D_{\Omega_2} = 4.0, \quad D_{\Omega_3} = 8.0 \tag{S2}$$

1.2 Additional results: Assessment of accuracy and robustness on a synthetic test case, NPH1

We will in this section present additional results that was not presented in the main text Sec. 2.2. The convergence was monitored for a wide range of parameters, some shown in Figure S5, and we saw the similar convergence for ADC and boundary conditions for $\alpha \in (10^{-6}, 10^{-4})$ and $\beta \in (10^{-4}, 1)$. Furthermore, the method was found robust with respect to the noise as illustrated in the Figure S1.

The variation in the number of observations was tested with 5,10 and 20 evenly spaced observations over the course of 24 hours. This was done together with 10 times steps, 20 time steps and 40 time steps. Illustrative results are shown in Figure S4, which shows that a high number of time steps compared to observations causes oscillations (e.g. purple line) at the boundary for $\alpha \gg \beta$. This is counteracted by selecting high values of the temporal regularization β relatively to α hereby enforcing smoothness (e.g. blue line). For example, in the geometry with grey and white matter, it was observed that the ADC error increased by a factor ≈ 4 for $\alpha \sim \beta$ relative to the case where $\beta \gg \alpha$ for the case with 20 time steps and 10 observations.

1.3 MRI contrast agent concentration - image signal relation

Below, we briefly describe the relationship between the imaging signal seen in Figure 2 and the underlying MRI contrast agent concentration. We remark that we use notations that are common in medical literature, which includes the use of two letter symbols. Hence, we will also use two letter symbols, such as TE and TR, to keep the notation consistent with the presentation in[1,2]. The MRI contrast agent concentration c causes the longitudinal(spin-lattice) relaxation time T_1 to shorten with the following relation

$$\frac{1}{T_1^c} = \frac{1}{T_1^0} + r_1 c. \tag{S3}$$

The superscripts indicate relaxation time with MRI contrast agent T_1^c and without MRI contrast agent T_1^0, while r_1 is the relaxivity constant for the MRI contrast agent in a medium. The MRI observations were obtained using a MRI sequence known as Magnetization Prepared Rapid Acquisition Gradient Echo (MPRAGE) with an inversion prepared magnetization. The relation between the signal and the relaxation time is non-linear, and is expressed with the following equations. The MRI signal value S for this sequence can be expressed as

$$S = M_n \sin\theta e^{-TE/T_2^*}, \tag{S4}$$

with TE and θ respectively denoting the echo time and the flip angle, and M_n is the magnetization for the n-echo that we described below. Also T_2^* is the transverse magnetization caused by a combination of spin-spin relaxation and magnetic field inhomogeneity, defined as

$$\frac{1}{T_2^*} = \frac{1}{T_2} + \gamma \Delta B_{in}. \tag{S5}$$

Here T_2 is the transverse (spin-spin) relaxation time, γ is the gyromagnetic ratio and ΔB_{in} is the magnetic field inhomogeneity across a voxel. The expression (S4) can be simplified by neglecting the exponential term, since $TE << T_2^*$ is a general trait for this MRI sequence. Thus, (S4) becomes

$$S = M_n \sin\theta. \tag{S6}$$

Magnetization for the n-echo M_n is defined as[1]:

$$M_n = M_0 \left[(1-\beta)\frac{(1-(\alpha\beta)^{n-1}}{1-\alpha\beta} + (\alpha\beta)^{n-1}(1-\gamma) + \gamma(\alpha\beta)^{n-1}\frac{M_e}{M_0} \right] \tag{S7}$$

with

$$\frac{M_e}{M_0} = -\left[\frac{1-\delta+\alpha\delta(1-\beta)\frac{1-\alpha\beta^m}{1-\alpha\beta}+\alpha\delta(\alpha\beta)^{m-1}-\alpha^m\rho}{1+\rho\alpha^m}\right]. \tag{S8}$$

Using the following definitions

$$\begin{aligned} \alpha &= \cos(\theta) \\ \beta &= e^{-T_b/T_1^c} \\ \delta &= e^{-T_a/T_1^c} \\ \gamma &= e^{-T_w/T_1^c} \\ \rho &= e^{-TR/T_1^c} \\ T_w &= TR - T_a - T_b(m-1). \end{aligned} \tag{S9}$$

Here T_b is known as the echo spacing time, T_a is the inversion time, T_w the time delay, TR as the repetition time, m is the number of echoes and M_0 is a calibration constant for the magnetization. The center echo denoted as $n = m/2$ will be the signal that we will consider when estimating concentration of the MRI contrast agent. Given (S6), the relative signal increase can be written as

$$\frac{S^c}{S^0} = \frac{M_n^c \sin(\theta)}{M_n^0 \sin(\theta)}. \tag{S10}$$

We define that

$$f(T_1) = M_n/M_0. \tag{S11}$$

Figure S2 shows $f(T_1)$ in CSF, grey and white matter. This gives the following relation

$$\frac{f(T_1^c)}{f(T_1^0)} = \frac{S^c}{S^0} \tag{S12}$$

The signal difference between observation times were adjusted in[3]. Thus we can express the change in T_1 due to MRI contrast agent as

$$f(T_1^c) = \frac{S^c}{S^0} f(T_1^0) \tag{S13}$$

and then estimate the concentration using (S3). The T_1^0 values were obtained by T1 mapping of the brain using a MRI sequence known as MOLLI5(3)3[4]. This takes into account patient specific characteristic, such as tissue damage. Tissue damage can be observed in the MRI due to a lower signal in the white matter compared to healthy white matter tissue, thus damaged tissue have different T_1 relaxation time. The MRI contrast agent concentration was estimated in a preprocessing step, using the parameters obtained from the T1-map, MPRAGE MRI protocol[3] and the value for r_1 found in[5]. The values of the function (S11) was computed for $T_1 \in (200, 4000)$ creating a lookup table. The lookup table was utilized with the baseline signal increase to estimate T_1^c, and then the concentration was computed using (S3).

1.4 Diffusion tensor imaging

DTI images provide apparent diffusion coefficients (ADC) for water molecules (18Da) on short time-scales. A processed DTI image is shown in Figure S3 with the largest ADC (shown in red in the middle figure) to be around 1.3e−3mm^2/s.

In order to compare the computed diffusion coefficient, we need to estimate the ADC for gadobutrol (604 Da)[6] by utilizing (1) together with the tortuosity and the free diffusion coefficient for gadobutrol. The tortuosity in the white and grey matter was estimated using (1) with the ADC obtained from DTI and the self-diffusion of water which is 3.0×10^{-3}mm^2/s at 37^oC[7]. We used the free diffusion coefficient for Gd-DPTA (550 Da)[8], which was reported in[9] to be 3.8×10^{-4}mm^2/s, as a surrogate for gadobutrol. This is based on the Stokes-Einstein equation, which states that molecules with similar weight will

have similar diffusion coefficients[10]. Furthermore, we need to assume that the tortuosity is independent for molecules with mass lower than 1kDa, i.e. not large proteins. The fractional anisotropy is defined as

$$FA^2 = \frac{3}{2}\frac{(\lambda_1 - MD)^2 + (\lambda_2 - MD)^2 + (\lambda_3 - MD)^2}{\lambda_1^2 + \lambda_2^2 + \lambda_3^2}, \tag{S14}$$

with the mean diffusivity MD, closely related to ADC, defined as

$$MD = \frac{\lambda_1 + \lambda_2 + \lambda_3}{3}. \tag{S15}$$

In these equations λ_i denotes the eigenvalues of the diffusion tensor. FA is a common measure of anisotropy and shown in the right-most image in Figure S3 for NPH1.

References

1. Gowland, P. & Leach, M. Fast and accurate measurements of T1 using a multi-readout single inversion-recovery sequence. *Magn. resonance medicine* **26**, 79–88 (1992).
2. Wang, J., He, L., Zheng, H. & Lu, Z.-L. Optimizing the magnetization-prepared rapid gradient-echo (MP-RAGE) sequence. *PloS one* **9**, e96899 (2014).
3. Ringstad, G. *et al.* Brain-wide glymphatic enhancement and clearance in humans assessed with MRI. *JCI insight* **3** (2018).
4. Taylor, A. J., Salerno, M., Dharmakumar, R. & Jerosch-Herold, M. T1 mapping: basic techniques and clinical applications. *JACC: Cardiovasc. Imaging* **9**, 67–81 (2016).
5. Rohrer, M., Bauer, H., Mintorovitch, J., Requardt, M. & Weinmann, H.-J. Comparison of magnetic properties of MRI contrast media solutions at different magnetic field strengths. *Investig. radiology* **40**, 715–724 (2005).
6. National Center for Biotechnology Information. Pubchem compound database; cid=15814656. https://pubchem.ncbi.nlm.nih.gov/compound/15814656. Accessed: 2018-10-13.
7. Le Bihan, D. & Johansen-Berg, H. Diffusion MRI at 25: exploring brain tissue structure and function. *Neuroimage* **61**, 324–341 (2012).
8. National Center for Biotechnology Information. Pubchem compound database; cid=6857474. https://pubchem.ncbi.nlm.nih.gov/compound/6857474. Accessed: 2018-10-13.
9. Gordon, M. J. *et al.* Measurement of Gd-DTPA diffusion through PVA hydrogel using a novel magnetic resonance imaging method. *Biotechnol. Bioeng.* **65**, 459–467 (1999).
10. Valencia, D. P. & González, F. J. Understanding the linear correlation between diffusion coefficient and molecular weight. a model to estimate diffusion coefficients in acetonitrile solutions. *Electrochem. Commun.* **13**, 129–132 (2011).

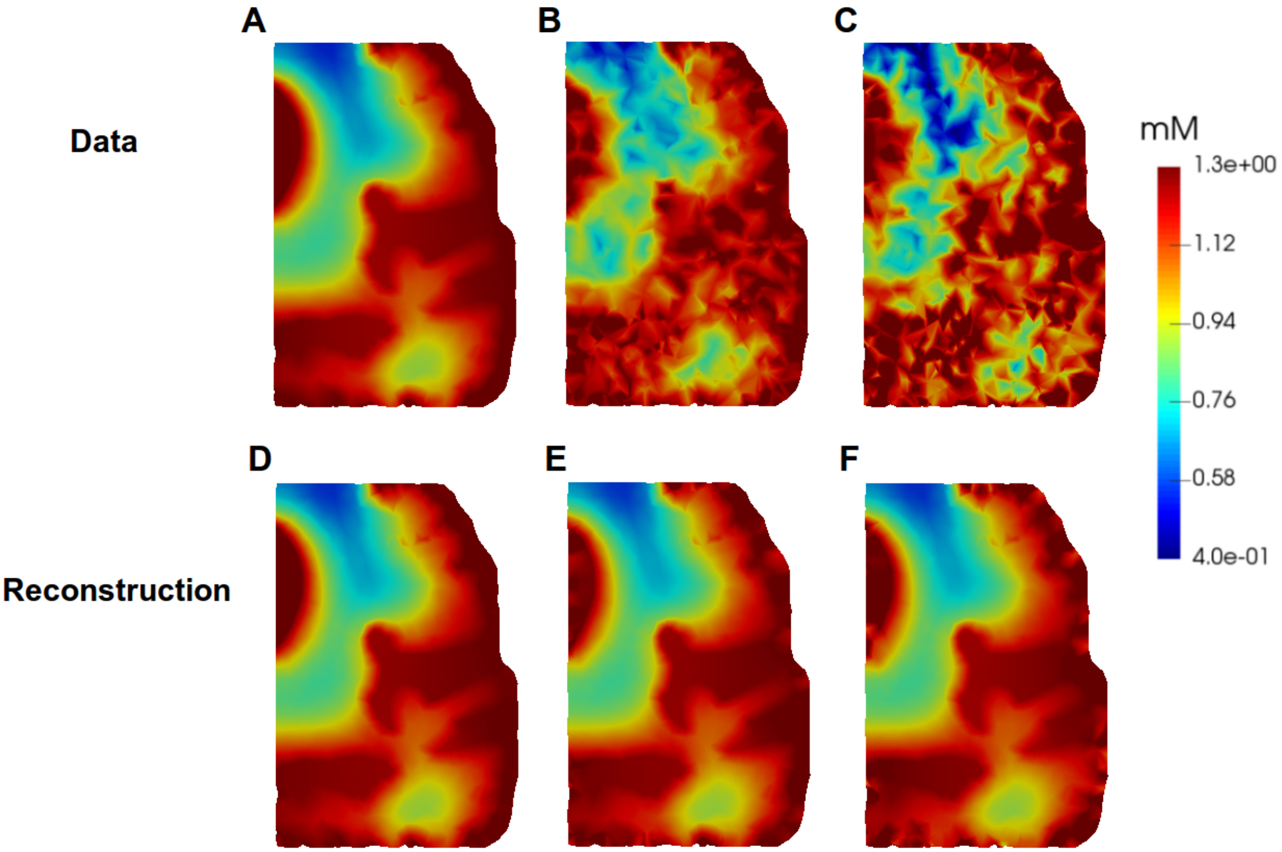

Figure S1. The upper row shows the manufactured observation in NPH1, A) Shows the manufactured observation at time-point 24 with no noise added. B) Shows the manufactured observation after 12 hours with noise range of (-0.15,0.15). C) Shows the manufactures observation after 12 hours with noise range of (-0.3,0.3). The lower row shows the results with optimized parameter obtained with $\alpha = 0.0001$, $\beta = 1.0$ and $k = 20$. D) Shows the resulting state given the observation in A. E) Shows the resulting state given the observation in B .F) Shows the resulting state given the observation in C.

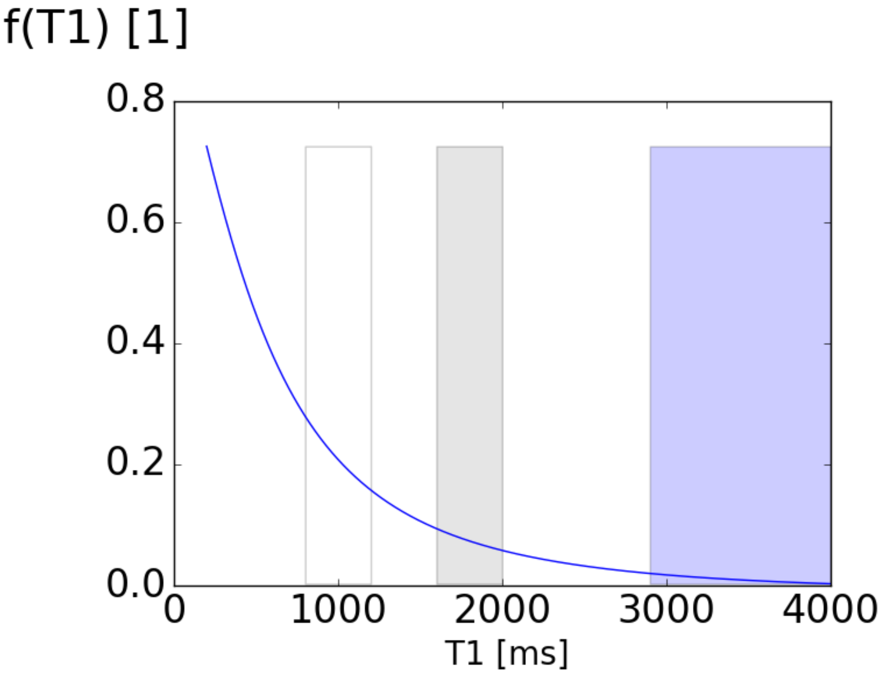

Figure S2. The image shows the function defined in (S11) where the white region indicates T_1 values for white matter, the grey region indicates T_1 values for grey matter, the blue region indicates T_1 values for CSF.

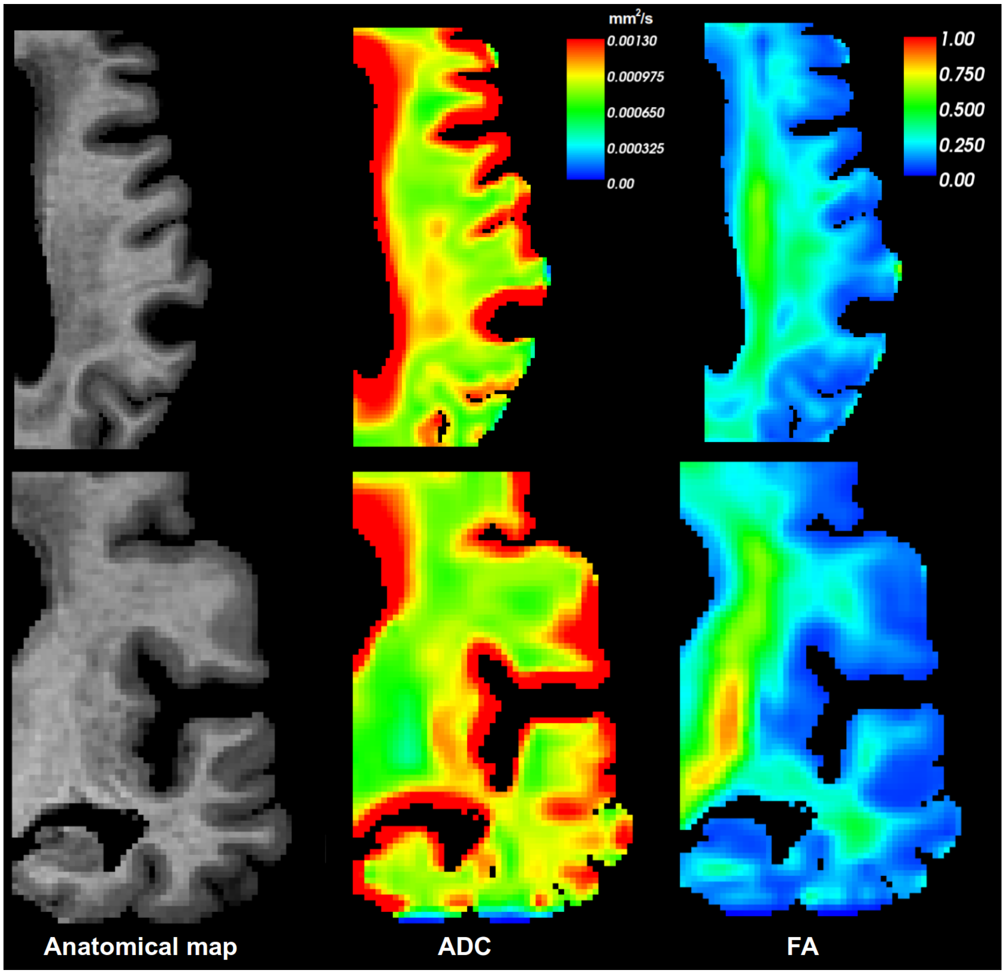

Figure S3. The left panel shows the anatomical map of NPH1. The middle panel shows the apparent diffusion coefficients (ADC) obtained from DTI. The right panel shows the computed fractional anisotropy (FA) from the DTI at a color coded scale.

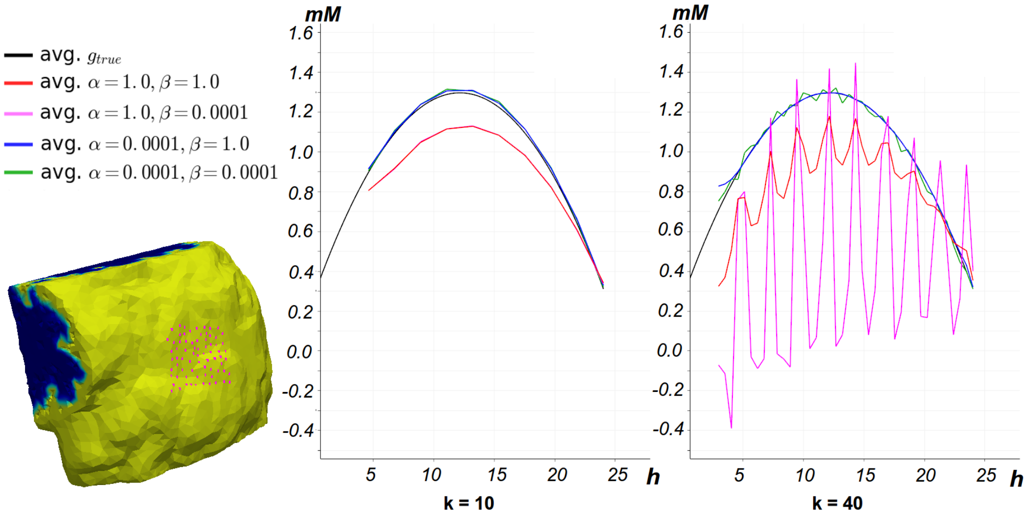

Figure S4. The image displays plots over time for a selection of points at the boundary of Ω_1 with different regularization parameters and number of time steps k. The left panel shows the legend for the plot over time, together with the selection of points. The middle panel shows the average boundary value g for different regularization parameter with $k = 10$. The right panel shows the average boundary value g for different regularization parameter with $k = 40$.

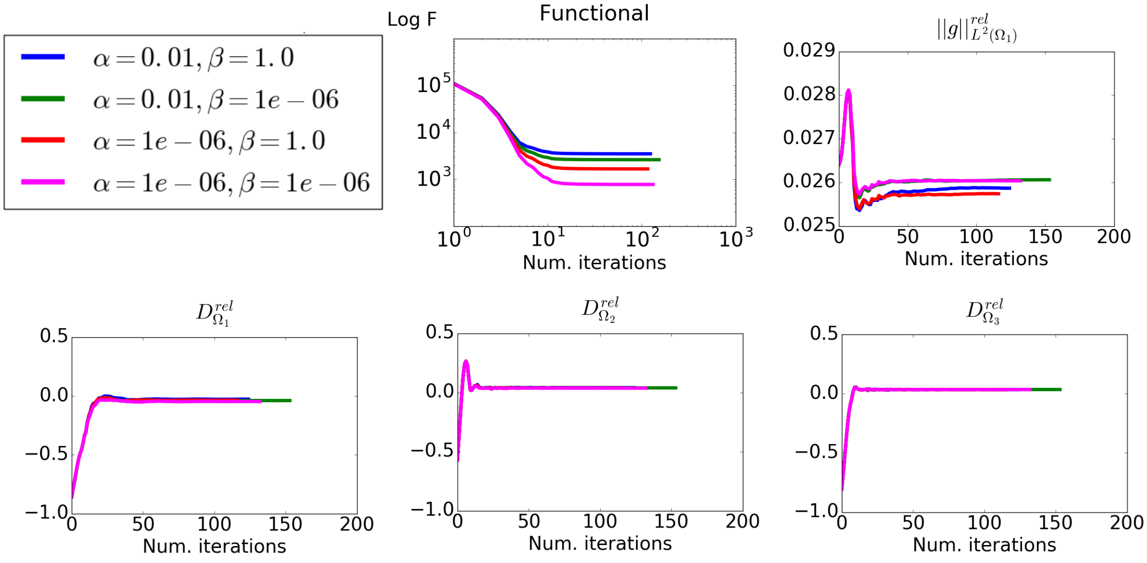

Figure S5. Convergence plots of the diffusion coefficients, boundary conditions and functional (2) with respect to different α and β values.
